# Clusterization of co-morbidities and multi-morbidities among persons living with HIV: a cross-sectional study

**DOI:** 10.1186/s12879-019-4184-z

**Published:** 2019-06-25

**Authors:** Paolo Maggi, Carmen R. Santoro, Marco Nofri, Elena Ricci, Nicolò De Gennaro, Chiara Bellacosa, Elisabetta Schiaroli, Giancarlo Orofino, Barbara Menzaghi, Antonio Di Biagio, Nicola Squillace, Daniela Francisci, Francesca Vichi, Chiara Molteni, Paolo Bonfanti, Giovanni Battista Gaeta, Giuseppe Vittorio De Socio

**Affiliations:** 1 Infectious Diseases Clinic University of Campania “Luigi Vanvitelli”, Neaples, Italy; 2 Infectious Diseases Clinic, University of Bari, Bari, Italy; 3 Infectious Diseases Clinic, Department of Medicine 2, Azienda Ospedaliera di Perugia and University of Perugia, Santa Maria Hospital, Perugia, Italy; 4 Department of Women, Child and Neonate, Fondazione IRCCS Ca’ Granda Ospedale Maggiore Policlinico, Milan, Italy; 5 Division I of Infectious and Tropical Diseases, ASL Città di Torino, Torino, Italy; 6 Unit of Infectious Diseases, ASST della Valle Olona, Busto Arsizio, VA Italy; 7 Infectious Diseases, San Martino Hospital Genoa, Genoa, Italy; 8 Infectious Diseases Unit ASST-MONZA, San Gerardo Hospital-University of Milano-Bicocca, Monza, Italy; 9 Infectious Diseases Clinic, “Santa Maria” Hospital, University of Perugia, Terni, Italy; 10 Infectious Diseases Unit, Santa Maria Annunziata Hospital, Usl centro, Florence, Italy; 11 Unit of Infectious Diseases, A. Manzoni Hospital, Lecco, Italy

**Keywords:** HIV, Co-morbidity, Multi-morbidity, Disease-disease interactions, Clusterization

## Abstract

**Background:**

Among people living with HIV (PLWH), the prevalence of non-HIV related co-morbidities is increasing. Aim of the present study is to describe co-morbidity and multi-morbidity, their clustering mode and the potential disease-disease interactions in a cohort of Italian HIV patients.

**Methods:**

Cross-sectional analysis conducted by the *Coordinamento Italiano per lo Studio di Allergia e Infezioni da HIV* (CISAI) on adult subjects attending HIV-outpatient facilities. Non-HIV co-morbidities included: cardiovascular disease, diabetes mellitus, hypertension, oncologic diseases, osteoporosis, probable case of chronic obstructive pulmonary disease (COPD), hepatitis C virus (HCV) infection, psychiatric illness, kidney disease. Multi-morbidity was defined as the presence of two or more co-morbidities.

**Results:**

One thousand and eighty-seven patients were enrolled in the study (mean age 47.9 ± 10.8). One hundred-ninety patients (17.5%) had no co-morbidity, whereas 285 (26.2%) had one condition and 612 (56.3%) were multi-morbid. The most recurrent associations were: 1) dyslipidemia + hypertension (237, 21.8%); 2) dyslipidemia + COPD (188, 17.3%); 3) COPD + HCV-Ab+ (141, 12.9%). Multi-morbidity was associated with older age, higher body mass index, current and former smoking, CDC stage C and longer ART duration.

**Conclusions:**

More than 50% of PLHW were multi-morbid and about 30% had three or more concurrent comorbidities. The identification of common patterns of comorbidities address the combined risks of multiple drug and disease-disease interactions.

## Background

In recent years, the prevalence of co-morbidities among Persons Living With HIV infection (PLWH) increased, as a consequence of aging, chronic inflammation, systemic immune activation, and long-term exposure to the combination antiretroviral therapy (ART) [1–3]. For this reason, PLWH had an increased use of non-antiretroviral drugs as compared to the general population [4]. Higher attention was recently paid to the treatment and the consequences of the main co-morbidities: cardiovascular [5], renal [6], neurocognitive [7, 8], bone disease [9], non-AIDS related cancers [10].

Managing patients with multiple chronic co-morbidities is more challenging than managing those with a single condition. Current HIV guidelines [11] recommend close monitoring of cardiovascular, metabolic, hepatic, renal, and bone health. Investigating common patterns and disease-disease interactions between co-morbidities could support the development of intervention models, improve the management of multi-morbid patients, and provide indication for prevention of multiple comorbidities in PLWH [12].

An important issue is how the co-morbidities associate among them (i.e. clusterization of co-morbidities), because of the implications in terms of disease-disease and drug-drug interactions. In fact, if the therapy recommended for one disease is contraindicated in presence of a concurrent medical condition, the usefulness of clinical practice guidelines is limited. In such a scenario, evidence-based treatment guidelines, designed for single diseases, can lead to serious therapeutic conflicts, due to disease-disease and drug-drug interactions. Management of multi-morbid patients is challenging, especially in emergency conditions.

To provide information on the issue, this cross-sectional study aimed at describing the pattern of co-morbidity and multi-morbidity in age class, their clustering mode and the potential disease-disease interactions in a group of PLWH referring to a network of Italian Infectious Diseases centers.

## Methods

This study is a cross-sectional analysis of baseline data from a cohort study, conducted by the *Coordinamento Italiano per lo Studio di Allergia e Infezioni da HIV* (CISAI). Patients analyzed were enrolled in the STOPS-HIV Study cohort, from July 2014 to December 2016: this is an ongoing cohort study including adult (≥18 years old) PLWH attending the participating centers. The participation was almost complete, with more than 99% of patients agreeing to enter the study. In September 2018, in the frame of this study, the Cluster Project collected additional information from clinical records of these patients, in order to investigate co-morbidities present at the time of enrollment.

Non-AIDS/non-HIV co-morbidities considered included: previous cardiovascular disease (pCVD), defined as previous diagnosis of myocardial infarction, stroke, angina pectoris, coronary artery, bypass grafting, angioplasty; diabetes mellitus, diagnosed with laboratory data and drug-tracing criteria [13]; high blood pressure, diagnosed with blood pressure measurement and drug-tracing criteria [14]; history of non-AIDS defining cancers, based on medical history, oncologic follow up and drug-tracing criteria; osteoporosis, based on bone density scan and drug-tracing criteria; clinical probable chronic obstructive pulmonary disease (COPD), based on the presence of at least 3 criteria among current smoking habit, age > 40 years, chronic cough, sputum production and dyspnea [15]; antibodies for hepatitis C (HCV Ab+) coinfection; psychiatric illness, based on medical history, specialist evaluation and drug-tracing criteria; kidney disease, based on estimated glomerular filtration rate (eGFR) < 60 ml/min, calculated according to the Modification of Diet in Renal Disease formula [16]; dyslipidemia, based on serum cholesterol (> 200 mg/dL) and triglycerides (> 150 g/dL) values and drug-tracing criteria [17].

Multi-morbidity was defined as the presence of two or more co-morbidities other than HIV infection. The criterion of two or more chronic conditions has been considered a cut-off score to compare multi-morbid and non multi-morbid groups.

All study participants provided informed consent to participate in the parent study, which was approved by the institutional ethics committee of the coordinating center (July 2014).

The STOPSHIV Study aimed at enrolling 1000 subjects. In the Cluster Project, this sample size was considered adequate in order to obtain a precision of at least 5% for any morbidity with prevalence ranging from 10 to 90%.

*Statistical methods.* Continuous data are presented as means (and standard deviation, SD), or medians (and interquartile range, IQR), and categorical data as frequency and proportions (%). We compared continuous data using analysis of variance test or Wilcoxon test and categorical data using chi-square statistics. Association between risk factors and multi-morbidity was assessed using the odds ratios (OR) and corresponding 95% Confidence intervals (CI), including in the unconditional logistic regression terms for age and sex. If a variable had less than 5% of missing value they were not included in the analysis. No information was missing in more than 5% of subjects.

We assumed a significance level at a *p*-value < 0.05. All statistical analyses were conducted using SAS 9.4 (SAS Institute, Inc., Cary, North Carolina).

## Results

A total of 1087 patients have been enrolled. 799 (73.5%) were men (mean age 48.6 ± SD 11.1), 288 (26.5%) women (46.0 ± 9.6); overall mean age was 47.9 ± 10.8. Patients were mainly Caucasians (89.2%); the main risk factor for HIV acquisition was heterosexual intercourse (44.7%). Other demographics are shown in Table 1.

**Table 1:** Characteristics of 1087 patients enrolled in the Cluster Project

Categorical variables		
	*N* = 1087	%
Gender		
Women	288	26.5
Men	799	73.5
Caucasian	1010	92.9
Current smoker	561	51.6
Former smoker	209	19.2
HIV risk factor		
MSM	299	27.5
IVDU	240	22.1
Heterosexual intercourse	486	44.7
Blood transfusion	7	0.6
Other	55	5.1
CDC Stage		
A	495	45.5
B	294	27.0
C	278	25.6
missing	20	1.8
ART naive	43	4.0
Cardiovascular events before enrolment	49	4.5
Multi-morbidity	612	56.3
Continuous variables	Mean or median	SD or IQR
Age (years)	47.9	10.8
BMI (Kg/m^2^)	24.7	4.1
Nadir CD4 (cells/mm^3^)	200.5	80.0–328.0
CD4 (cells/mm^3^)	634.0	430.0–824.0
Years since ART initiation	9.0	4.0–16.0
Serum creatinine (mg/dL)	0.92	0.63
Glycemia (mg/dL)	93.0	20.9
Total cholesterol (mg/dL)	186.1	41.5
Triglycerides (mg/dL)	118	84–178
eGFR (mL/min)	97.3	27.0

*SD* standard deviation, *IQR* interquartile range, *MSM* men having sex with men, *IVDU* intravenous drug user, *BMI* body mass index, *ART* antiretroviral treatment, *eGFR* estimated glomerular filtration rate

**Fig. 1:**
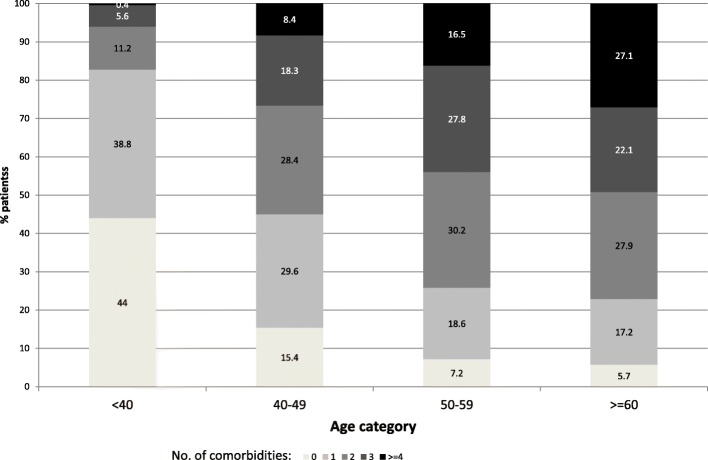
Multi-morbidity prevalence by age categories. The following morbidity were included: CVD events, diabetes mellitus, hypertension, dyslipidemia, HCV Ab+, psychiatric illness, osteopenia/osteoporosis, renal impairment

One hundred-ninety patients (17.5%) had no co-morbidity, whereas 285 (26.2%) had one condition and 612 (56.3%) were multi-morbid: 25.3% had two co-morbidities, 19.3% three, 8.2% four,3.5% five or more. The most frequent co-morbidity was dyslipidemia (55.3%), followed by hypertension (31.4%), COPD (29.4%), hepatitis C virus (HCV) infection (25.4, 5.5% with detectable HCVRNA), psychiatric illness (10.3%), diagnosis of osteopenia/osteoporosis (10.1%), diabetes (6.1%), and renal impairment (4.8%); 95 (8.7%) subjects had history of non-AIDS-defining cancer. Forty-nine patients (4.5%) had pCVD events. Distribution of comorbidities by age class is shown in Fig. 1.

**Fig. 2:**
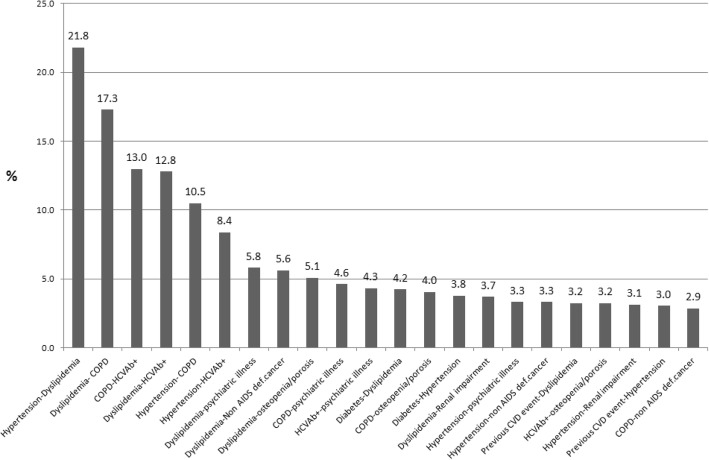
Most frequent pairs of comorbidities

Analyzing the pairs of co-morbidities, the most recurrent associations were: 1) dyslipidemia + hypertension (237, 21.8%); 2) dyslipidemia + COPD (188, 17.3%); 3) COPD + HCV-Ab+ (141, 12.9%). Other frequent associations are shown in Fig. 2.

When we compared parameters of HIV infections and patients’ characteristics between multi-morbid and non multi-morbid patients, after adjusting for age and sex, we observed that all variables were significantly related with multi-morbidity status, except for CD4 cell count used as a continuous variable (by 100 cells/mm^3^); being ART naïve was not significantly different between groups, although the proportion of ART naïve patients was lower in the multi-morbid group (Table 2). Multi-morbid patients were older and showed lower nadir CD4 cell count, longer period in ART therapy, and, as expected since these variables were used for comorbidity definition, had higher triglycerides, glycemia, and total cholesterol (Table 2). They were more frequently in CDC stage B and C than in stage A. In a model including age, sex, body mass index (BMI), cigarette smoking, intravenous drug use (IVDU) as risk factor for HIV acquisition, CDC stage, ART duration (by 2 years), and current CD4 < 200 cells/mm^3^, multi-morbidity was associated to CDC stage, years of ART, and current CD4 count < 200 cells/mm^3^. In the multivariable analysis, type of ART regimen, whether based on protease inhibitors (PI), non-nucleoside reverse transcriptase inhibitors (NNRTI) or integrase strand transfer inhibitors (INSTI), did not significantly differ between groups.

**Table 2:** Comparison of patients’ characteristics and parameters of HIV infections, between multi-morbid and non multi-morbid patients. Adjusted odds ratios for risk of multi-morbidity

	Multi-morbidity *N* = 612 (56.3%)	No multi-morbidity *N* = 475 (43.7%)	aOR 95% CI
Age, years, mean ± SD	51.7 ± 9.1	42.9 ± 10.7	1.08 (1.06–1.09) by 1 year
Women, N (%)	136 (22.2%)	152 (32.0%)	0.81 (0.58–1.15) ref. men
BMI, Kg/m^2^, mean ± SD	25.2 ± 4.6	24.0 ± 3.2	1.17 (1.07–1.16) by 1 point
Smoking, N (%)			
Never	113 (18.5%)	204 (43.0%)	1
Current	265 (43.3%)	195 (41.0%)	3.65 (2.54–5.24)
Former	133 (21.7%)	76 (16.0%)	1.77 (1.14–2.75)
Risk factor for HIV acquisition IVDU	203 (33.2%)	37 (7.8%)	3.19 (2.02–5.04) ref. other risk factors
CDC stage, N (%)			
A	229 (37.4%)	266 (56.0%)	1
B	177 (28.9%)	117 (24.6%)	1.20 (0.83–1.74)
C	195 (31.9%)	83 (17.5%)	2.04 (1.38–3.02)
ART duration, years, median (IQR)	12 (6–17)	6 (2–11)	1.08 (1.02–1.13) by 2 years
Current CD4, < 200 cells/mm^3^, N (%)	42 (6.9%)	23 (4.8%)	1.97 (1.01–3.87) ref. ≥200 cells/mm^3^
ART naïve, N (%)	14 (2.3%)	29 (6.1%)	1.32 (0.56–3.10) ref. experienced
Nadir CD4, cells/mm^3^, median (IQR)	170 (70–290)	242 (105–364)	1.00 (0.96–1.05) by 50 cells/ mm^3^
CD4, cells/mm^3^, median (IQR)	636 (434–852)	623 (425–780)	1.02 (0.97–1.08) by 100 cells/ mm^3^
Current PI, N* (%)	272 (44.4%)	169 (35.6%)	1.16 (0.84–1.59) ref. no PI
Current NNRTI, N* (%)	238 (38.9%)	172 (36.2%)	1.07 (0.78–1.47) ref. no NNRTI
Current INSTI, N* (%)	179 (29.2%)	108 (22.7)	1.22 (0.86–1.75) ref. no INSTI
N. of drugs in ART regimen			
0	27 (4.4%)	56 (11.8%)	ref.
1	24 (3.9%)	40 (8.4%)	0.72 (0.28–1.82)
2	89 (14.5%)	38 (8.0%)	1.72 (0.76–3.93)
3	447 (73.0%)	323 (68.0%)	1.24 (0.63–2.44)
>=4	25 (4.1%)	18 (3.8%)	1.33 (0.50–3.53)
			χ2 for trend *p* = 0.39
Glycemia, mg/dL, mean ± SD	96.3 ± 25.3	88.8 ± 12.0	1.06 (1.01–1.12) by 5 mg/dL
Total cholesterol, mg/dL, mean ± SD	192.2 ± 42.9	178.3 ± 38.2	1.05 (1.03–1.08) by 5 mg/dL
Triglycerides, mg/dL, median (IQR)	146 (98–201)	96 (70–128)	1.08 (1.05–1.10) by 10 mg/dL

*SD* standard deviation, *IQR* interquartile range, *aOR* adjusted odds ratio, *CI* confidence interval, *BMI* body mass index, *IVDU* past intravenous drug use, *ART* antiretroviral treatment; all variables are adjusted (as appropriate) for age (continuous variable), sex, BMI (continuous variable), cigarette smoking, IVDU, CDC stage, ART duration (by 2 years), current CD4 count (< 200 cells/mm^3^), except for naïve status, that was only adjusted for age, sex, BMI, smoking habit, current CD4 cells count, and current CD4 count as a continuous variable, that was adjusted for age (continuous variable), sex, BMI (continuous variable), cigarette smoking, IVDU, CDC stage, and ART duration (by 2 years)

The main factors associated with multi-morbidity were: age, BMI and smoking with CVD/COPD multi-morbidity; age, smoking, and IVDU with CVD/HCV Ab; smoking and CDC stage with CVD/psychiatric illness; age and CDC stage with CVD/non-AIDS-defining cancer; age, smoking, IVDU and CDC stage with CVD/osteopenia or osteoporosis.

**Fig. 3:**
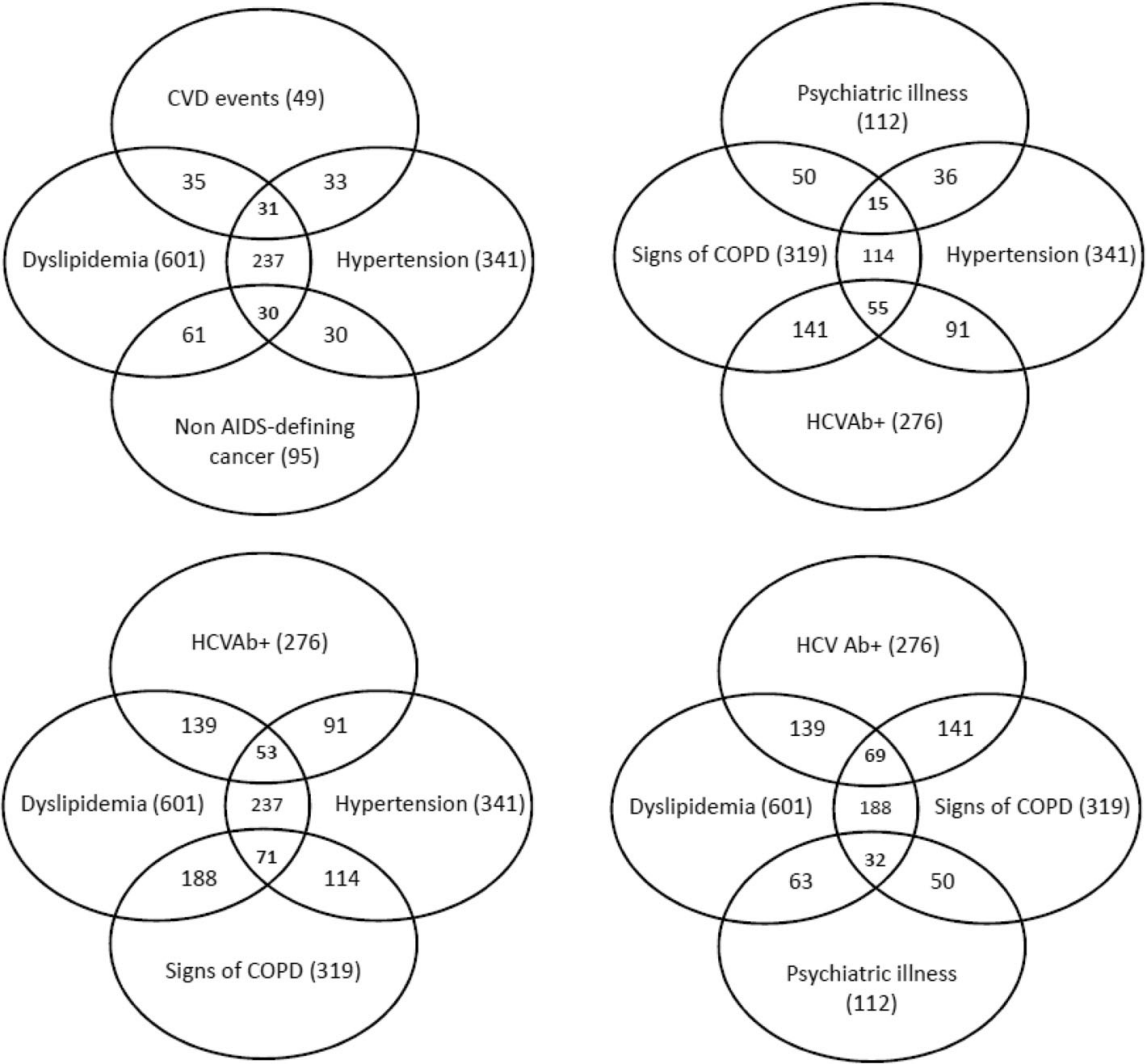
Most frequent triads of comorbidities

Recombining the co-morbidities in triads (Fig. 3), we observed that the most recurrent ones were: 1) dyslipidemia + hypertension + COPD (71, 6.6%), significantly associated with age (aOR 1.06, 95% CI 1.03–1.09 by 1 year) and BMI (aOR 1.10, 95% CI 1.04–1.16 by 1 point); 2) dyslipidemia + COPD + HCV-Ab+ (69 patients, 6.3%), more frequent in patients with longer ART duration (aOR 1.18, 95% CI 1.08–1.28 by 2 years of treatment); 3) hypertension + COPD + HCV-Ab+ (55, 5.1%), significantly associated with sex (aOR 3.13, 95% CI 1.36–7.22) and years of ART (aOR 1.20, 95% CI 1.10–1.32 by 2 years); 4) dyslipidemia + hypertension + HCV (53, 4.9%), related to BMI (aOR 1.08, 95% CI 1.01–1.15), current smoking (aOR 4.88, 95% CI 1.68–14.10) and ART duration (aOR 1.19, 95% CI 1.08–1.31 by 2 years).

We also had details on drug treatments for dyslipidemia and hypertension. Out of 601 subjects with dyslipidemia, 102 (17.0%) were on statins, and 58 (9.6%) on other lipid-lowering drugs (some patients on both), thus leaving 76.0% of subjects without treatment. Older patients (≥65 years old) were more likely be treated than younger ones (61.5% vs 20.4%, *p* < 0.0001). Similarly though slightly better, out of 341 patients with high blood pressure, 208 (61.0%) were on antihypertensive drugs and 133 (39.0%) without treatment: 74.1% of hypertensive subjects ≥65 years old were on treatment, versus 58.5% of those aged less than 65 years (*p* = 0.03).

## Discussion

In our sample of patients attending the HIV-outpatient facilities in the participating centers, we found that most PLHW were multi-morbid and about 30% had three or more concurrent conditions. Besides other known risk factors, shared with the general population, such as age, BMI and cigarette smoking, we found that CDC stage, duration of ART and current CD4 cell count < 200 cells/mm^3^ were risk factors for multi-morbidity.

As regards the frequency of two or more concurrent medical conditions and their clusterization, data are scarce and difficult to compare, at least partly because of heterogeneous definitions of multi-morbidity [18, 19]. In particular, few studies were performed among PLWH. However, our findings are largely consistent with the results of recent studies [20–22].

In a cross-sectional study comparing 208 HIV positive subjects with 208 matched HIV negative controls [20], the frequency of multi-morbidity was higher in the former (63% vs 43%) than in the latter, and associated with duration of HIV and ART. In our sample, multi-morbid patients were about 54%, a lower proportion likely due to the younger age of enrolled subjects, and infection variables (duration of ART and advanced CDC stage) were similarly linked to higher probability of having 2 or more co-morbidities.

Comparing co-morbidity in a large cohort of veterans with and without HIV infection, Goulet et al. [21] found that co-morbidity was the rule (60% in HIV positive veterans), and multi-morbidity was common among those with HIV infection (10%). Patterns of comorbidity differed substantially by HIV status, age, and HIV severity. In multivariable analyses, older HIV-infected veterans were more likely to have substance use disorder and multi-morbidity.

Kim et al. [22] performed a study on a population of PLWH, examining the relationship with obesity and multi-morbidity. They found that prevalence increased with progressive BMI categories. Three multi-morbidity clusters were identified: “Metabolic”; “Behavioral”; and “Substance Use”. Obesity was associated with a higher likelihood of multi-morbidity: consistently, our findings show a higher prevalence of multi-morbidity with higher BMI, even after accounting for potential confounders: this result was led by the positive association between BMI and diabetes, hypertension and dyslipidemia, even if a significant inverse relationship was observed with HCV and osteopenia/osteoporosis.

In Italy, comorbidity prevalence has been evaluated by Guaraldi et al. [23] in a sample of 2854 PLWH: using similar diagnostic criteria, previous CVD event and renal impairment prevalence estimates were largely similar, but we found a much lower proportion of subjects with diabetes (6.1% versus 11.1%) and a higher proportion of hypertensive patients (31.4% versus 22.2%). In our study as well as in Guaraldi et al.’s, age was the main determinant of multi-morbidity, although we did not find a significant detrimental role of being male. This discrepancy may be due to the inclusion of smoking and past IVDU in our analysis: in our sample, both factors were more frequent in men than in women, and had a significant relationship with multi-morbidity. Multi-morbidity prevalence could not be compared between studies as reported, because it depended on considered diseases: however, if we defined multi-morbidity as Guaraldi et al. did, just considering diabetes, bone diseases (although with different definition), renal impairment and hypertension, our prevalence estimate was similar (about 14%).

In Geriatric HIV patients older than 65 years from Italy (GEPPO cohort) multi-morbidity was largely present (64% of cases) [24]. Although patients older than 65 years represented only a small proportion (6.3%) of our sample, we consistently found that multi-morbidity was present in about 76.7% of cases (56/73). Also, we observed that in this subgroup drug treatment for hypertension and dyslipidemia was more frequent than in younger PLWH, probably because the concern for CVD risk overrode the risk of drug-drug interaction, in clinicians’ opinion.

In a recent paper, comorbidities were assessed in PLWH participating in two independent cohorts: POPPY and AGEhIV [12]. Analysis identified 6 patterns among the 1073 POPPY: CVD, sexually transmitted diseases, mental health problems, cancers, metabolic disorders, chest/other infections. The CVD pattern was positively associated with cancer, while the mental health pattern was correlated with all the other patterns In the 598 AGEhIV 6 patterns were identified: CVDs, chest/liver, HIV/AIDS events, mental health/neurological problems, STDs, and general health. The general health pattern was correlated with all the other patterns.

Interestingly, Markun et al. [25] performed a study, including HIV negative subjects, with the aim to identify therapeutic conflicts in emergency department patients with multi-morbidity. In this paper, major therapeutic conflict was defined as a situation where clinical practice guidelines recommended a treatment of a medical condition, that is absolutely contraindicated because of a coexisting condition (for example, a situation where anticoagulation is recommended because of a pulmonary embolism, but at the same time contraindicated because of a co-existing gastrointestinal bleeding). Minor therapeutic conflict was defined as a situation where clinical practice guidelines recommended a treatment of one medical condition, that is relatively contraindicated because of a co-existing condition, but the treatment is possible without adverse effects if certain precautions are taken (for example, a situation where acetylsalicylic acid is recommended because of a vascular disease, but at the same time contraindicated because of a co-existing reflux esophagitis). Among major conflicts they found: cancer requiring chemotherapy or immunosuppression in patients with renal failure, or with cirrhosis at risk of gastrointestinal bleeding; and CVD requiring acetylsalicylic acid or oral anticoagulants in cirrhotic patients with disturbances of coagulation. Among minor conflicts they found: CVD requiring diuretic treatment in patients with renal failure; CVD requiring antihypertensive agents in patients with renal failure or with cirrhosis at risk of gastrointestinal bleeding; CVD in patients requiring beta blockers in patients with asthma. Most of these disease-disease interactions are not infrequent in PLWH, but we are not aware of similar studies among these patients.

We also had information on drug treatment for hypertension and dyslipidemia. In both cases, we noted that these conditions were undertreated, with about 25% of dyslipidemic patients on any lipid-lowering treatments, and 60% of hypertensive subjects on hypotensive drugs, as already observed in samples of Italian patients [26, 27]. These two conditions were part of the most frequent triads of non-AIDS/non-HIV related conditions observed in our study, and as such they remained mostly untreated. This finding may be related both to patients’ reluctance to take other drugs, being subjects to a substantial pill burden for HIV, and to physicians’ concern of potential drug-drug interactions, in the context of the chronic treatment for HIV infection.

Our data evidence that, in spite of mean age lower than 50, co-morbidity was the rule among our PLWH (82%), and that more than 50% of our patients were multi-morbid. Moreover, about 30% of them had three or more chronic non-HIV related conditions, thus confirming recent data provided by other studies in the field [8]. Dyslipidemia was, by far, the most frequent co-morbidity, but potential HCV-related damage is still a concern, mainly in patients with long-lasting co-infection, despite the big efforts under way to cure this condition. In the light of the study of Markun et al. [25], the chronic conditions that appear most at risk for disease-disease interactions are liver disease, renal impairment and cardiovascular diseases. In our experience, both in pairs and in triads they appear frequently associated with other comorbidities, and among them. All the conditions potentially at risk for major and minor disease-disease interactions suggested by Markun [22] (respectively cancer + renal impairment, cancer + liver disease, CVD + liver disease, and CVD + renal impairment, CVD + liver disease, CVD + COPD) were present among our patients, although in different percentages. Not unexpectedly, multi-morbid patients presented higher mean age, worse parameters of HIV infections, more compromised metabolic data and renal function with respect to non multi-morbid ones.

In our study we found that cigarette smoking, a modifiable risk factor, was significantly associated with multi-morbidity. This lifestyle factor is linked to increased morbidity and mortality for both HIV and non-HIV related diseases: tobacco use and HIV together may accelerate the development of chronic obstructive pulmonary disease, lung cancer and death [28, 29], and smoking resulted associated with a higher risk of myocardial infarction in the HIV-infected than in the general population as well [30]. Our findings emphasize the need of smoking cessation strategy, with the expectation of longer life expectancy and better quality of life.

The main limitation of our study is the absence of a control group of HIV-negative subject. Moreover, the investigation aimed at identifying only the potential disease-disease interactions; in particular, we considered all the HCVAb+ patients, regardless of the level of liver decompensation. Similarly, we considered also risk factors for CVD, not only the presence of overt cardiac disease. Even if our objective was not to examine polypharmacy, a further challenge should be to evaluate the impact of disease-disease interactions on polypharmacy and, consequently, on drug-drug interactions. Few studies address the problem of clusterization of chronic non communicable conditions among PLWH and, although other cohorts examined a higher number of patients respect to our experience, data were extracted from diagnosis codes, like ICD-9-CM [21], or from electronic health records [22]. In our study, data were provided by the infectious disease specialists who followed their patients, thus providing more reliable and updated data. As a main strength of this study, we could account for several factors potentially related to multi-morbidity, among infection variables, patients’ characteristics and lifestyle factors, primarily smoking habit.

## Conclusions

Considering the aging of PLWH, their persistent exposure to chronic inflammation and the possible effects of HAART, it is mandatory to address the phenomenon of clusterization timely, and to detect all the patients at risk of disease-disease interactions. The evaluation of common patterns of comorbidities in PLWH may help to identify and address the combined risks of major and minor therapeutic conflicts and their potential impact on adverse effects and drug-drug interactions, as we previously evidenced and exemplified. Moreover, this should help to adequate the clinical practice guidelines recommendation to this important example on how the “medicine of complexities” is rapidly emerging in our daily clinical practice.

## Data Availability

The dataset analyzed during the current study is available from the corresponding author on reasonable request.

## Abbreviations

AIDS: Acquired Immunodeficiency Syndrome
aOR: adjusted odds ratio
ART: Antiretroviral therapy
BMI: Body mass index
CDC: Center for Diseases Control
CI: Confidence interval
CISAI: Coordinamento Italiano per lo Studio di Allergia e Infezioni da HIV
COPD: Chronic obstructive pulmonary disease
CVD: Cardiovascular diseases
eGFR: estimated glomerular filtration rate
HCV Ab+: antibodies for hepatitis C virus
HIV: Human Immunodeficiency Virus
INSTI: Integrase strand transfer inhibitors
IQR: Interquartile range
IVDU: Intravenous drug use
NNRTI: Non nucleoside reverse transcriptase inhibitors
OR: Odds ratio
PI: Protease inhibitors
PLWH: Persons living with HIV
pCVD: previous cardiovascular disease
SD: Standard deviation

## References

[1] Krentz HB, Kliewer G, Gill MJ (2005). Changing mortality rates and causes of death for HIV-infected individuals living in southern Alberta, Canada, from 1984 to 2003. HIV Med.

[2] Grinzstejn B, Luz PM, Pacheco AG, Santos DV, Velasque L, Moreira RI (2013). Changing mortality profile among HIV-infected patients in Rio de Janeiro, Brazil: shifting from AIDS to non-AIDS related conditions in the HAART era. PLoS One.

[3] Ford N, Shubber Z, Meintjes G, Grinsztejn B, Eholie S, Mills EJ (2015). Causes of hospital admission among people living with HIV worldwide: a systematic review and meta-analysis. Lancet HIV.

[4] Rasmussen LD, Kronborg G, Larsen CS, Pedersen C, Gerstoft J, Obel N, Pottegård A (2017). Use of non-antiretroviral drugs among individuals with and without HIV-infection: a Danish nationwide study. Infect Dis (Lond).

[5] Maggi P, Di Biagio A, Rusconi S, Cicalini S, D'Abbraccio M, d'Ettorre G (2017). Cardiovascular risk and dyslipidemia among persons living with HIV: a review. BMC Infect Dis.

[6] Rasch MG, Engsig FN, Feldt-Rasmussen B, Kirk O, Kronborg G, Pedersen C (2012). Renal function and incidence of chronic kidney disease in HIV patients: a Danish cohort study. Scand J Infect Dis.

[7] Madsen LW, Fabricius T, Hjerrild S, Hansen TM, Mössner BK, Birkemose I (2014). Depressive symptoms are frequent among drug users, but not associated with hepatitis C infection. Scand J Infect Dis.

[8] van den Dries LWJ, Wagener MN, Jiskoot LC, Visser M, Robertson KR, Adriani KS, van Gorp ECM (2017). Neurocognitive impairment in a chronically well-suppressed HIV-infected population: the Dutch TREVI cohort study. AIDS Patient Care STDs.

[9] Kruger MJ, Nell TA (2017). Bone mineral density in people living with HIV: a narrative review of the literature. AIDS Res Ther.

[10] Ji Y, Lu H (2017). Malignancies in HIV-infected and AIDS patients. Adv Exp Med Biol.

[11] European AIDS Clinical Society (EACS) Guidelines. Version 9.1 October 2018. Available online at: http://www.eacsociety.org/files/2018_guidelines-9.1-english.pdf

[12] De Francesco D, Verboeket SO, Underwood J, Bagkeris E, Wit FW, Mallon PWG (2018). Pharmacokinetic and clinical observations in PeoPle over fiftY (POPPY) study and the AGEhIV cohort study. Patterns of co-occurring comorbidities in people living with HIV. Open Forum Infect Dis.

[13] American Diabetes Association (2016). Classification and diagnosis of diabetes. Diabetes Care.

[14] Mancia G, Fagard R, Narkiewicz K, Redón J, Zanchetti A, Böhm M (2013). Task force members. 2013 ESH/ESC guidelines for the management of arterial hypertension: the task force for the management of arterial hypertension of the European Society of Hypertension (ESH) and of the European Society of Cardiology (ESC). J Hypertens.

[15] Rabe KF, Hurd S, Anzueto A, Barnes PJ, Buist SA, Calverley P (2007). Global strategy for the diagnosis, management, and prevention of chronic obstructive pulmonary disease: GOLD executive summary. Global initiative for chronic obstructive lung disease. Am J Respir Crit Care Med.

[16] Levey AS, Bosch JP, Lewis JB, Greene T, Rogers N, Roth D (1999). A more accurate method to estimate glomerular filtration rate from serum creatinine: a new prediction equation. Modification of diet in renal disease study group. Ann Intern Med.

[17] National Cholesterol Education (2002). Program detection, evaluation and treatment of high blood cholesterol in adults (adult treatment panel III). Circulation..

[18] Fortin M, Stewart M, Poitras ME, Almirall J, Maddocks H (2012). A systematic review of prevalence studies on multi-morbidity: toward a more uniform methodology. Ann Fam Med.

[19] Valderas J, Starfield B, Sibbald B, Salisbury C, Roland M (2009). Defining comorbidity: implications for understanding health and health services. Ann Fam Med.

[20] Maciel RA, Klück HM, Durand M, Sprinz E (2018). Comorbidity is more common and occurs earlier in persons living with HIV than in HIV-uninfected matched controls, aged 50 years and older: a cross-sectional study. Int J Infect Dis.

[21] Goulet JL, Fultz SL, Rimland D, Butt A, Gibert C, Rodriguez-Barradas M (2007). Do patterns of comorbidity vary by HIV status, age, and HIV severity?. Clin Infect Dis.

[22] Kim DJ, Westfall AO, Chamot E, Willig AL, Mugavero MJ, Ritchie C (2012). Multi-morbidity patterns in HIV-infected patients: the role of obesity in chronic disease clustering. J Acquir Immune Defic Syndr.

[23] Guaraldi G, Orlando G, Zona S, Menozzi M, Carli F, Garlassi E (2011). Premature age-related comorbidities among HIV-infected persons compared with the general population. Clin Infect Dis.

[24] Guaraldi G, Malagoli A, Calcagno A, Mussi C, Celesia BM, Carli F (2018). The increasing burden and complexity of multi-morbidity and polypharmacy in geriatric HIV patients: a cross sectional study of people aged 65 - 74 years and more than 75 years. BMC Geriatr.

[25] Markun S, Holzer BM, Rodak R, Kaplan V, Wagner CC, Battegay E (2014). Therapeutic conflicts in emergency department patients with multi-morbidity: a cross sectional study. PLoS One.

[26] De Socio GV, Ricci E, Parruti G, Calza L, Maggi P, Celesia BM (2016). Statins and aspirin use in HIV-infected people: gap between European AIDS clinical society guidelines and clinical practice: the results from HIV-HY study. Infection.

[27] De Socio GV, Ricci E, Maggi P, Parruti G, Pucci G, Di Biagio A (2014). CISAI study group. Prevalence, awareness, treatment, and control rate of hypertension in HIV-infected patients: the HIV-HY study. Am J Hypertens.

[28] Sigel K, Wisnivesky J, Gordon K (2012). HIV as an independent risk factor for incident lung cancer. AIDS..

[29] Reddy KP, Kong CY, Hyle EP (2017). Lung Cancer mortality associated with smoking and smoking cessation among people living with HIV in the United States. JAMA Intern Med.

[30] Rasmussen LD, Helleberg M, May MT (2015). Myocardial infarction among Danish HIV-infected individuals: population-attributable fractions associated with smoking. Clin Infect Dis.

